# A Single-Sensor Approach to Quantify Gait in Patients with Hereditary Spastic Paraplegia

**DOI:** 10.3390/s23146563

**Published:** 2023-07-20

**Authors:** Linda M. A. van Gelder, Tecla Bonci, Ellen E. Buckley, Kathryn Price, Francesca Salis, Marios Hadjivassiliou, Claudia Mazzà, Channa Hewamadduma

**Affiliations:** 1 Department of Mechanical Engineering, INSIGNEO Institute for In Silico Medicine, The University of Sheffield, Sheffield S10 2TN, UK; linda.vangelder@nhs.net (L.M.A.v.G.); e.e.buckley@sheffield.ac.uk (E.E.B.); c.mazza@sheffield.ac.uk (C.M.); 2 Academic Department of Neurosciences, Sheffield Teaching Hospitals NHS Trust, University of Sheffield, Sheffield S10 2TN, UK; kitty_price@outlook.com (K.P.); m.hadjivassiliou@nhs.net (M.H.); chewamadduma1@sheffield.ac.uk (C.H.); 3 Department of Biomedical Sciences, University of Sassari, 07100 Sassari, Italy; fsalis1@uniss.it; 4 The Sheffield Institute for Translational Neuroscience (SITraN), University of Sheffield, Sheffield S10 2TN, UK

**Keywords:** hereditary spastic paraplegia, gait analysis, wearables, inertial sensor

## Abstract

Hereditary spastic paraplegia (HSP) is characterised by progressive lower-limb spasticity and weakness resulting in ambulation difficulties. During clinical practice, walking is observed and/or assessed by timed 10-metre walk tests; time, feasibility, and methodological reliability are barriers to detailed characterisation of patients’ walking abilities when instrumenting this test. Wearable sensors have the potential to overcome such drawbacks once a validated approach is available for patients with HSP. Therefore, while limiting patients’ and assessors’ burdens, this study aims to validate the adoption of a single lower-back wearable inertial sensor approach for step detection in HSP patients; this is the first essential algorithmic step in quantifying most gait temporal metrics. After filtering the 3D acceleration signal based on its smoothness and enhancing the step-related peaks, initial contacts (ICs) were identified as positive zero-crossings of the processed signal. The proposed approach was validated on thirteen individuals with HSP while they performed three 10-metre tests and wore pressure insoles used as a gold standard. Overall, the single-sensor approach detected 794 ICs (87% correctly identified) with high accuracy (median absolute errors (*mae*): 0.05 s) and excellent reliability (ICC = 1.00). Although about 12% of the ICs were missed and the use of walking aids introduced extra ICs, a minor impact was observed on the step time quantifications (*mae* 0.03 s (5.1%), ICC = 0.89); the use of walking aids caused no significant differences in the average step time quantifications. Therefore, the proposed single-sensor approach provides a reliable methodology for step identification in HSP, augmenting the gait information that can be accurately and objectively extracted from patients with HSP during their clinical assessment.

## 1. Introduction

Hereditary spastic paraplegias (HSPs) are a group of heterogeneous neurodegenerative disorders characterised by progressive lower-limb spasticity resulting in gait disturbance. This occurs as a result of their pyramidal tract dysfunction [1]. In some clinics, the assessment of walking in people with HSP, as part of their routine in-clinic neurologic examinations, is usually limited to a timed test, such as a 10-m walk, to estimate their gait speed. A detailed and objective analysis of their gait pattern is usually not completed due to challenges regarding time, feasibility, and reliance on complex gait lab assessments. Therein, this is a missed opportunity to capture and fully characterise their gait pattern and how this might change over time. Objective quantifications of the gait pattern of people with HSP may help with the monitoring of gait, making decisions regarding skeletal muscle relaxants, and also the identification of biomarkers relevant to the disease [2].

Previous research that aimed to characterise gait in people with HSP primarily used three-dimensional gait analysis [2,3,4]. Based on these studies, HSP can be described as an overall reduced walking speed, cadence, stride length, and range of motion of the distal segments when compared to healthy controls [4,5]. Such gait features can be used to discriminate patients with HSP from patients with similar clinical characteristics, such as patients with cerebral palsy [2], sporadic spastic paraplegia [3], and spastic diplegia [4]. Therefore, validated gait-related biomarkers identified via gait analysis have the potential to be useful tools in predicting ambulatory natural history and clinical trial design and evaluating the treatment efficacy of novel treatments in HSP to help characterise, develop, and quantify the effect of treatment for these patients.

The parameters of stride characteristics appear clinically valid to reflect gait impairment in HSP [6]. Compared to healthy participants, patients with HSP have markedly higher stride times (13%), which have been shown to significantly correlate to the Spastic Paraplegia Rating Scale (SPRS) values, i.e., to the severity of HSP and to the Falls Efficacy Scale-International (FES-I) questionnaire, i.e., to the patient-reported fear of falling. A longitudinal multicentre clinical study with eleven participants found that after a mean interval of 14 months, there was a significant deterioration in the SPRS scores and gait speed and a significant increase in the stride time [6]. Therefore, the incorporation of objective gait measures into future clinical trial designs will help to characterise, develop, and quantify the efficacy of novel treatments for these patients. To facilitate and encourage the inclusion of such measurement tools, it is essential to reduce both the patients’ and clinicians’ burdens.

Small body-mounted devices, such as inertial measurement units (IMUs), are ideal tools to complement and/or replace three-dimensional gait analysis, which requires specialist gait laboratories. These wearable monitoring systems typically contain accelerometers, gyroscopes, and magnetometers and allow the accurate measurement of motion during various activities that are not constrained to a confined laboratory. Moreover, IMUs are quick to set up and affordable. IMUs placed on both feet of participants have shown promise in the measurement of mobility and provide clinically valid parameters that reflect mobility impairment in patients with HSP [6,7]. To further limit the burden on patients and clinicians, approaches exploiting the adoption of a single IMU device located on the pelvis are usually preferred to extract mobility outcomes [8]. Lower-back single-sensor algorithms have been proposed in healthy cohorts [9,10,11] and adapted for patients with different diseases, including multiple sclerosis, stroke, Parkinson’s disease, and hemiparesis and for older adults [12,13,14]. Generally, a sound technical validation is recommended when algorithms tailored for a given cohort are then adopted in patients characterised by different walking impairments [15,16]. However, to the authors’ knowledge, to date, similar lumbar single-sensor approaches have not been validated in patients with HSP. This is especially necessary due to the acceleration pattern heterogeneity that is present in this cohort as a result of their various movement impairments. This also highlights the need for ad-hoc methodological developments and more extensive clinical validation [17].

Focusing on foot-to-ground event identification, which represents the first essential step in the quantification of most gait metrics, the aim of this study was to assess the validity of a single-sensor approach compared to a reference wearable multi-sensor system. As the foot-to-ground event represents the first essential step in the quantification of most gait metrics, this work focuses on the identification of individual steps (i.e., foot-to-ground event detection) and quantifying their duration (i.e., the interval between events from the ipsi- and contralateral foot) in patients with HSP. Considering that step identification using a single-sensor approach can be particularly challenging in the presence of walking aids, the impact of their adoption was also assessed in our study.

## 2. Materials and Methods

### 2.1. Participants

This study was approved by the Yorkshire and Humber—Sheffield Research Ethics Committee (Ref: 19/YH/0221). A convenience sample of 13 participants was selected from those who currently undergo an instrumented gait analysis assessment as part of their standard clinical care in the dedicated Hereditary Spastic Paraplegia and Ataxia clinics at the Sheffield Teaching Hospitals Foundation, NHS Trust, UK. The participants’ characteristics can be found in Table 1. Three clinical rating scales were used: SPRS, which rates functional impairment in pure spastic paraplegia, with 0 being no dysfunction and 52 being most severe dysfunction; Modified Ashworth Scale (MAS, assessing muscle spasticity for eight muscles, where 0 refers to no increase in muscle tone and 4 refers to the affected part(s) rigid in flexion or extension; if 1+ was assigned, this was scored as 1.5); and the Scale for the Assessment and Rating of Ataxia (SARA, assessing ataxia symptom severity, ranging from 0 “no ataxia” to 40 “most severe ataxia”). Participants were included if they had a definitive diagnosis of HSP (with a confirmed genetic mutation in a known HSP-associated gene), were over 18 years old, were able to perform at least one 10-metre walk test with or without assistance, and had a shoe size 36 European Union (EU) (3 UK) or above. Participants showing any significant comorbidities affecting their walking abilities (i.e., recent surgery, significant lower-limb injuries) or not exhibiting floor clearance during the swing phase of their gait cycle were not included in the study.

### 2.2. Walking Protocol

Each participant was asked to perform a 10-metre walking test along a clear hospital corridor, where the start and end of the walkway were marked with lines of tape on the ground. The patients began the test standing with their toes behind the first line and when instructed, they walked the length of the walkway at their usual walking pace and stopped walking when the second line was cleared with both feet. The tests were repeated three times and the participants were allowed to take rest as needed. One participant was only able to perform the 10-metre test twice due to fatigue.

### 2.3. Measurement Systems

**Figure 1:**
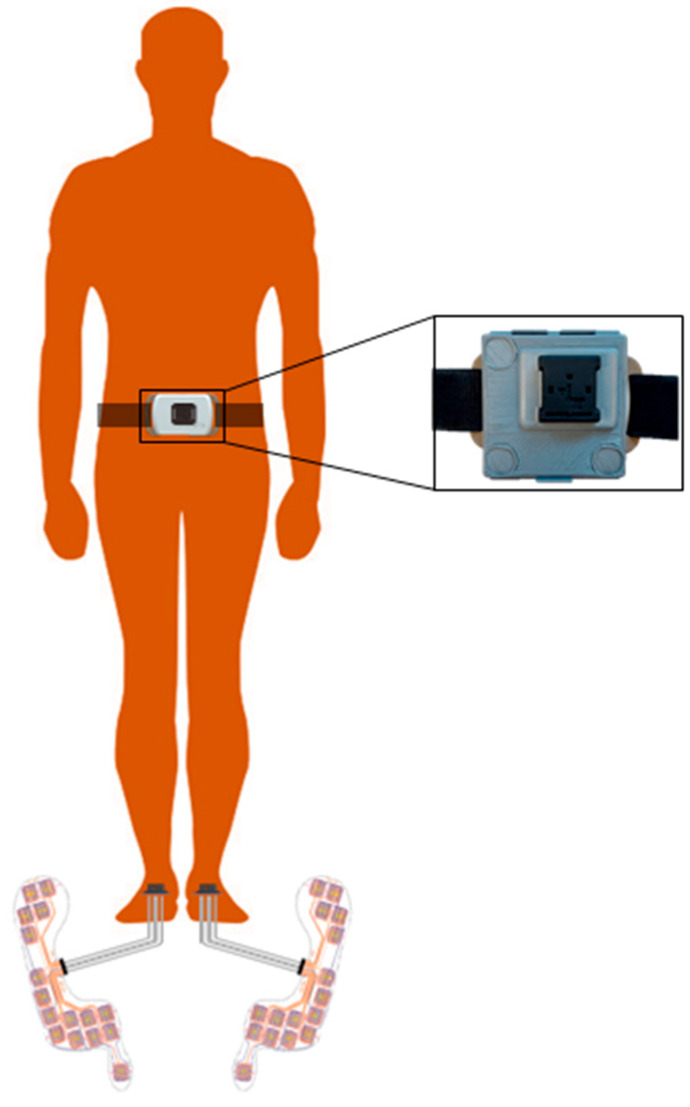
Adopted experimental setup for the validation of a single lower-back IMU (DynaPort MM+) for step detection in patients with hereditary spastic paraplegia. Pressure insoles were inserted into participants’ shoes to provide reference initial contacts. For data synchronization, an additional IMU unit (as part of the INDIP system) was rigidly fixed to the DynaPort MM+.

The participants were asked to simultaneously wear two systems: a multi-sensor system (INertial module (IMU) with DIstance Sensors and Pressure insoles, INDIP [18,19]) and a single IMU device (DynaPort MM+, McRoberts, The Hague, The Netherlands, dimensions: 106.6 × 58 × 11.5 mm, size: 55 g, sampling frequency 100 Hz) placed on the lower back via an elastic Velcro strap. The INDIP included two plantar pressure insoles (PIs, 16 force resistive sensing elements, fs = 100 Hz, manufacturer 221e S.r.l., Abano Terme, Italy), which were fitted inside the patient’s shoes and used as a reference system to validate the gait events extracted from the single lower back IMU. To be able to synchronise the data captured with the two systems (INDIP and DynaPort MM+), in addition to a common timestamp vector, an IMU from the INDIP system was also placed on the lower back. The two IMUs (IMU INDIP and IMU DynaPort MM+) were rigidly fixed together using a 3D-printed case (Figure 1). The magnitude of the gyroscope signal was calculated for each of the two lumbar sensors and, using the cross-correlation between these two signals, the delay was used to synchronise the data.

### 2.4. Data Processing

The reference foot–ground initial contacts ($IC_{PI}$) were identified from the PIs using a cluster-based approach [20]. Specifically, a first derivative approach was used to identify rising minima, which were used as reference points. Then, for each possible $IC_{PI}$, a subgroup of three rising minima was selected, corresponding to the activation of neighbouring sensors. An $IC_{PI}$ corresponded to the third rising minimum of the subgroup [20].

The ICs for the IMU DynaPort MM+ ($IC_{IMU}$) were calculated based on the detection of positive zero-crossings identified from the lower back 3D acceleration based on the peak enhancement procedure proposed by Paraschiv-Ionescu et al. [21]. The acceleration resultant ($ACC_{R}$) was calculated as:

(1) $$ACC_{R}=\sqrt{Acc_{AP}{}^{2}+Acc_{ML}{}^{2}+Acc_{V}{}^{2}}$$

**Figure 2:**
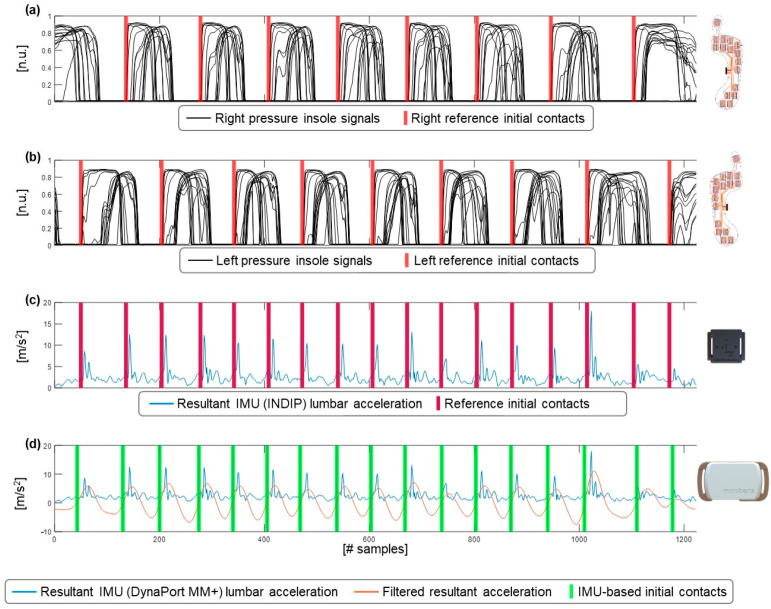
(**a**) Right reference initial contacts based on pressure signals (normalised units [n.u.]) of the right insole, (**b**) Left reference initial contacts based on pressure signals (normalised units [n.u.]) of the left insole, (**c**) Reference initial contacts based on the pressure insoles, as shown in (**a**,**b**), displayed on the resultant acceleration signal [m/s^2^] of the lumbar IMU of the INDIP system, (**d**) Resultant acceleration [m/s^2^], filtered resultant acceleration [m/s^2^], and the initial contacts based on the zero-crossings of the filtered signal.

The acceleration resultant was then resampled based on the smoothness of the signal. The smoothness was defined as the root mean square of the resultant signal, including samples 100–600. The first 100 samples and the final samples were discarded to remove the gait initiation and gait termination phases of the walking bout. The signal was resampled at 40 Hz. However, when the smoothness was above 8 m/s^2^, the signal was resampled at 60 Hz and, if the 10-metre walk duration was greater than 3 min, the signal was resampled at 20 Hz. The resampled data were then filtered to further smooth the data using a linear Savitzky–Golay filter (order = 7, frame length = 21). The data were then detrended and low-pass filtered (FIR filter, n = 120 coefficients, Fc = 2–3 Hz), as described by Paraschiv-Ionescu et al. [21]. To further improve the signal-to-noise ratio and enhance the step-related peaks, the signal was then smoothed and differentiated using the continuous wavelet transform (cwt, scale 10, gauss2 wavelet), followed by a linear Savitzky–Golay filter (order = 5, frame length = 11) [17]. From the filtered acceleration signal, all the positive zero-crossings were selected as $IC_{IMU}$ (Figure 2).

### 2.5. Statistical Analysis

#### 2.5.1. Performance Metrics Based on Initial Contact Detection

Using the $IC_{PI}$ and a tolerance window of 0.5 s [22] centred on them, the $IC_{IMU}$ were labelled as true positives (TP), false positives (FP), and false negatives (FN); the additional $IC_{IMU}$ events detected outside the tolerance windows were labelled as FPs. The following performance metrics were then calculated for each participant and test:

(2) $$Sensitivity=\frac{\;TP}{TP+FN}$$

(3) $$Positive\;Predicted\;Value\;\left(PPV\right)=\frac{TP\;}{TP+FP}$$

(4) $$F1=2\times \frac{\;PPV\times Sensitivity\;}{\;PPV+Sensitivity}$$

#### 2.5.2. Accuracy

Considering the TP ICs detected for each participant and the walking test, the differences between the $IC_{IMU}$ and $IC_{PI}$ were calculated for each detected *i*-TP IC to establish the relevant time error:

(5) $$e_{IC,i}=IC_{IMU,i}-IC_{PI,i},\;i=1,\ldots ,\mathrm{TP}$$

To evaluate how $e_{IC,i}$ propagated on the step time values (T), which were determined by the duration between two consecutive ICs, the reference ($T_{PI}$) and IMU-based ($T_{IMU}$) step time values were assessed for the *n* detected steps during the recorded walking tests:

(6) $$e_{T,j}=T_{IMU,j}-T_{PI,j},\;j=1,\ldots ,n$$

Moreover, considering all the ICs detected with the IMU-based approach (i.e., TP, and FP), errors in the average step time ($\bar{T}$) were determined for each *k*-walking test:

(7) $$e_{\bar{T},k}={\bar{T}}_{IMU,k}-{\bar{T}}_{PI,k},\;k=1,\ldots ,38$$

The median error (*me*), median absolute error (*mae*), and interquartile range errors (*iqre*) were quantified for $e_{IC}$, $e_{T}$, and $e_{\bar{T}}$ to assess their relevant bias, accuracy, and precision [23], respectively.

For both the step time (T) and average step time ($\bar{T}$), the relative errors (e%) were quantified as:

(8) $$e{\%}_{T,j}=\left|\frac{T_{IMU,j}-T_{PI,j}}{T_{PI,j\;}}\right|\times 100,$$

(9) $$e{\%}_{\bar{T},k}=\left|\frac{{\bar{T}}_{IMU,k\;}-{\bar{T}}_{PI,k}}{{\bar{T}}_{PI,k\;}}\right|\times 100.$$

The relevant accuracy and precision were established as median and interquartile range relative values: *mae%* and *iqre%*. A Shapiro–Wilk test showed that the step time was not normally distributed and, therefore, non-parametric tests were used to compare the estimations obtained with the two systems.

#### 2.5.3. Reliability

As with the accuracy, the reliability was calculated based on a comparison of the ICs identified by both systems (i.e., including only true positive ICs). Furthermore, the reliability was calculated based on the average step time for each walking test for each participant in order to define the reliability of each test.

To assess the reliability between the systems, the intraclass correlation coefficient (ICC_2,1_) estimates and their 95% confidence intervals (CI) were calculated based on a single rater, absolute agreement, two-way random-effects model [24,25,26,27]. Values less than 0.5, between 0.5 and 0.75, between 0.75 and 0.9, and greater than 0.9 indicated poor, moderate, good, and excellent reliability, respectively [24]. A Wilcoxon signed-rank test (non-parametric data) was performed to define the significant differences based on a comparison of both systems. Finally, for the average step time, a Bland–Altman plot, calculation of the limits of agreement (LOA), and the correlation between $e_{\bar{T}}$ and the reference average step time were assessed.

#### 2.5.4. Effect of Using Walking Aids

For all the above-mentioned metrics, to gain insight into the effect of the use of walking aids, the cohort was divided into two subgroups depending on whether they completed the 10-metre walk test with (n = 7, three participants used one stick or cane, two used two crutches, and two used a rollator) or without a walking device (n = 7). One participant indeed completed one walking test with one stick, while the other two 10-metre tests were completed without a walking aid. Therefore, this one participant was part of both groups. The differences between the two subgroups (walks with or without a walking aid) were defined with the Mann–Whitney U test (non-parametric test). Given the reduced sample size, the effect size (r) for non-parametric tests was computed as r = $\frac{\left|z\right|}{\sqrt{N}}$, where z is the standardised z-score based on the adopted test as calculated in SPSS, and N is the number of total observations on which z is based. The thresholds were 0.1, 0.3, and 0.5, as recommended by Cohen [28,29] for small, medium, and large effect sizes, respectively. All the statistical analyses were performed using SPSS version 26 (SPSS Inc., Chicago, IL, USA).

## 3. Results

### 3.1. Initial Contact Detection—Performance Metrics

The data from a total of 38 walking tests were processed; 788 reference ICs were detected (i.e., $IC_{PI}$), while the single-sensor approach identified 794 ICs. Among them, 694 ICs were identified as true positives, 94 as false negatives, and 100 as false positives. Among the various walking tests, the analysis of the IC detection showed a median F1 score of 0.94 [IQR: 0.86, 0.97], with a sensitivity of 0.94 [IQR: 0.82, 0.96] and a positive predicted value of 0.94 [IQR: 0.88, 1.00].

**Figure 3:**
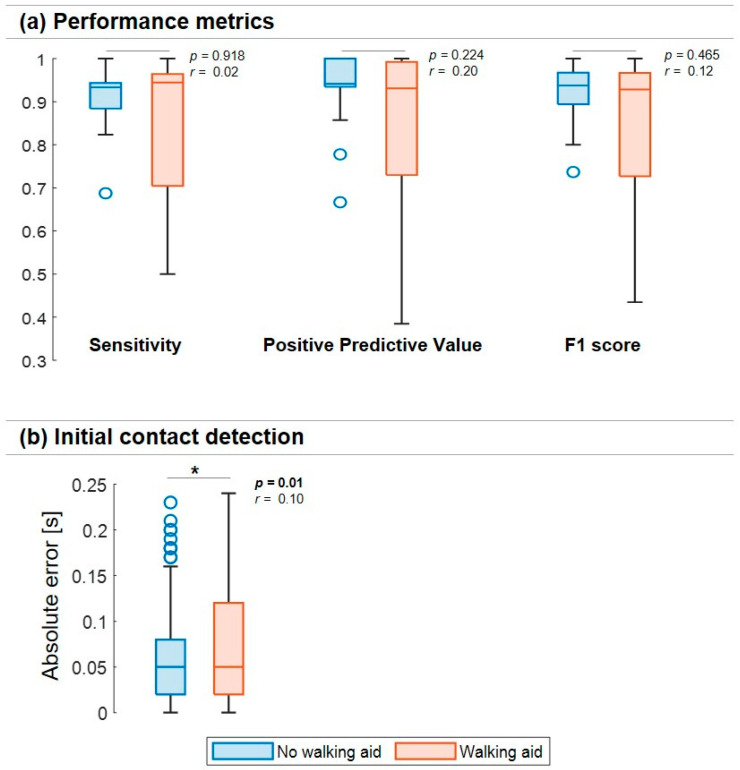
Comparison of patients with and without a walking aid for (**a**) different performance metrics (sensitivity, positive predictive value, and F1 score) and (**b**) absolute errors [s] in the initial contact detection. * shows a significant *p*-value. Boxplots (minimum, lower quartile, median, upper quartile, and maximum) are used to display values obtained from the different walking tests. Outliers are also shown.

Of the total 38 tests that were performed, 19 tests were performed without a walking aid and 19 were performed with a walking aid. The two subgroups contributed differently to the above overall errors, as shown by the IC detection performances shown in Figure 3a. However, no significant differences were found in the explored performance metrics when comparing the tests in which walking aids were used to the tests in which no walking aids were used (Figure 3a).

### 3.2. Initial Contact Detection—Accuracy

Overall, a −0.03 s IC bias (i.e., *me*) was observed, while *mae* and *iqre* were 0.05 s and 0.08 s [0.02–0.10 s], respectively. In the tests without walking aids, 307 TP ICs were identified, while 387 TP ICs were identified for the tests in which a walking aid was used. The tests with a walking aid (*me*: −0.04 s, *mae*: 0.05 s, *iqre:* 10 s [0.02–0.12 s]) had significantly higher absolute errors (*U* = 66,157.5, *p* = 0.01) with a small effect size compared to the tests without a walking aid (*me*: −0.02 s, *mae*: 0.05 s, *iqre*: 0.06 s [0.02–0.08 s], Figure 3b).

### 3.3. Initial Contact Detection—Reliability

The $IC_{IMU}$ had an excellent agreement with the $IC_{PI}$, with 95% confidence intervals ranging within excellent agreement (ICC_2,1_ = 1.00, 95% CI = 1.00–1.00) across all the participants. Based on the Wilcoxon signed-rank test, a significant difference was found between the estimates from the two approaches (z = −9.79, *p* < 0.001, r = 0.37), since $IC_{IMU}$ detection was anticipated (on average 33.7 ms) compared to the reference $IC_{PI}$. The use of walking aids differently affected the IC detection, which was further anticipated when a walking aid was used (on average 43.6 ms, CI: 35.1–52.0 ms) compared to non-walking aid participants (on average 21.6 ms, CI: 12.3–30.8 ms, U = 68,622.5, *p* < 0.001).

### 3.4. Step Time Accuracy

**Figure 4:**
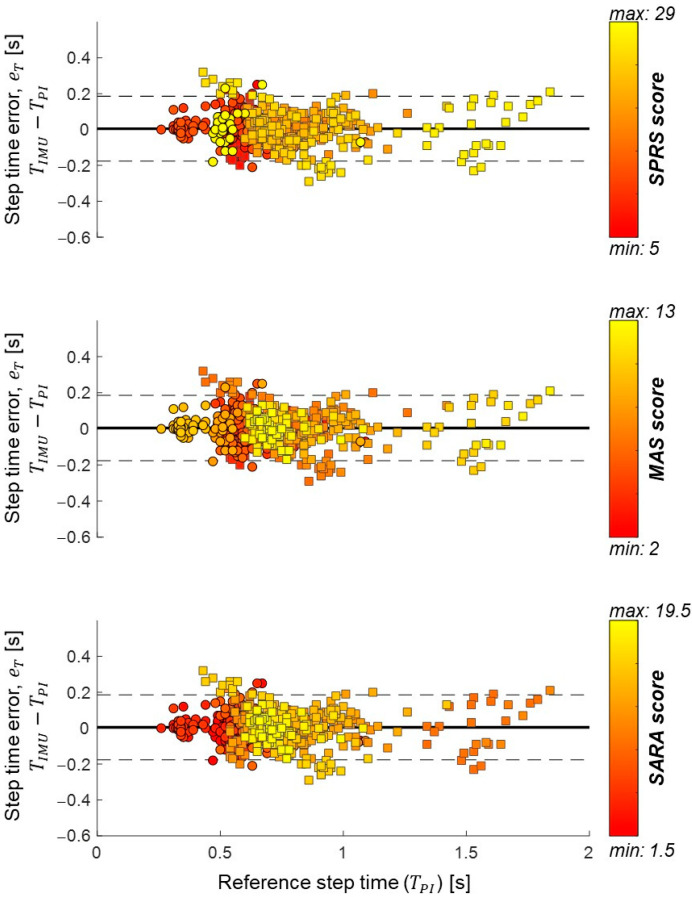
Bland–Altman plots for step time error values ($e_{T}$) calculated between IMU-based and reference step durations. Errors are assessed for each detected step (*n* = 596) and patients. Bias (continuous lines) and limit of agreements (dashed lines) are also shown. Values identified for steps performed by patients using walking aids are shown with squares; circles are used otherwise. The different colours indicate the disability severity based on the three clinical scales (colour-coded based on the relevant bar shown on the right): Spastic Paraplegia Rating Scale (SPRS), upper panel; Modified Ashworth Scale (MAS), middle panel; Scale for the Assessment and Rating of Ataxia (SARA), lower panel.

**Figure 5:**
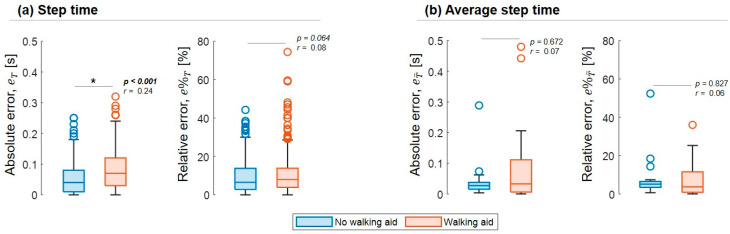
Boxplots (minimum, lower quartile, median, upper quartile, and maximum) of the absolute [s] and relative [%] errors for step time (**a**) and the average step time (**b**) duration distributions in patients with and without a walking aid are shown. Outliers are also shown. * shows a significant *p*-value.

In total, 596 steps were concurrently identified by both systems; a Wilcoxon signed-rank test showed that the single-sensor approach provided step time values that were statistically equivalent to those quantified with the reference one (Z = 1.260, *p* = 0.208, r = 0.05). Bland–Altman plots of $e_{T}$ are shown in Figure 4; the errors located farther apart from the LOA belonged to patients presenting the highest disability scores in at least one clinical scale. The reference step durations for the patients using walking aids were significantly longer (mean: 0.84 s, CI: 0.81, 0.87 s) than those observed in the other patients (mean: 0.58 s, CI: 0.57, 0.60 s, U = 74,406.5, *p* < 0.001). Although no statistical difference was observed in $e_{T}$ between the two groups (U = 43,612, *p* = 0.802), higher accuracy and precision were observed for the participants that did not require walking aids ($mae_{T}$*:* 0.04 s, $iqre_{T}$: 0.07 s) compared to walking aid users ($mae_{T}$*:* 0.08 s, $iqre_{T}$e: 0.09 s; U = 56,520.5, *p* < 0.001, Figure 5a). Conversely, there was no significant difference in the step time relative errors when exploring the effect of using a walking aid (Figure 5a).

A total of 38 walking tests were performed by the participants, and for each walking test, the relevant average step time was calculated. The median ${\bar{T}}_{IMU}$ based on the 38 tests was 0.71 s [IQR: 0.60 s, 0.80 s], and the median ${\bar{T}}_{PI}$ was 0.67 s [IQR: 0.57 s, 0.82 s]. The median absolute error $mae_{\bar{T}}$ was 0.03 s, $iqre_{\bar{T}}$: 0.05 s [0.01 s, 0.06 s], and the median relative error was $mae{\%}_{\bar{T}}$ = 5.09% and $iqre{\%}_{\bar{T}}$ = 7.96% [1.48%, 9.44%].

Of the total 38 tests that were performed, 19 tests were performed without a walking aid and 19 were performed with a walking aid. There were no significant differences in either the absolute or relative errors when exploring the effect of using a walking aid (Figure 5b).

### 3.5. Step Time Reliability

**Figure 6:**
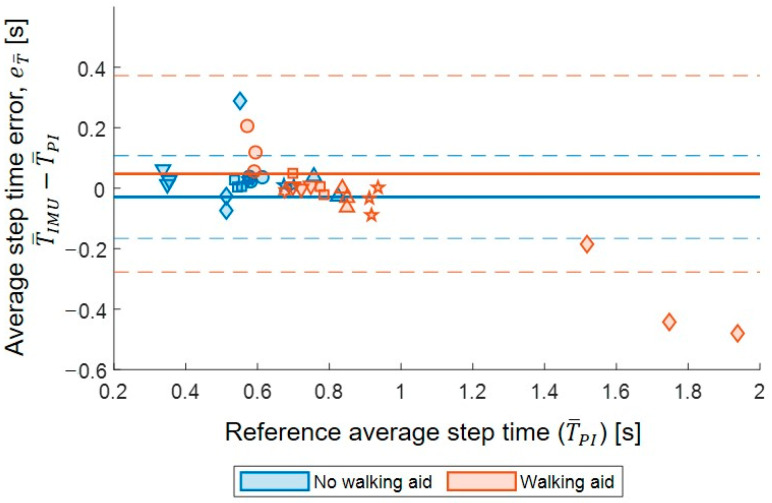
Bland–Altman plot for average step time ($\bar{T}$) error between ${\bar{T}}_{IMU}$ (single-sensor approach) and ${\bar{T}}_{PI}$ (reference pressure insole, reference): $e_{\bar{T}}$. Errors are assessed for each participant and walking test. Bias (continuous lines) and limit of agreement (dashed lines) are shown in blue for no walking aid and orange for walking aid users. The various symbols within the graph represent the different participants.

The average step times calculated from the IMU had excellent agreement with the average step times calculated from the PIs, with 95% confidence intervals ranging within excellent agreement (single measures, ICC_2,1_ = 0.899, 95% CI = 0.815–0.946) across all the participants. Based on the Wilcoxon signed-rank test, no significant differences were found between the estimates from the two systems (Z = −0.920, *p* = 0.357). Overall, the bias and limits of agreement were −0.01 s ± 0.26 s. A heteroskedastic distribution was observed, based on a negative statistically significant correlation between the average step time difference and the reference step time values (ρ = −0.601, *p* <0.001). The Bland–Altman plot comparing the average step time errors in the tests performed with and without a walking aid is shown in Figure 6.

## 4. Discussion

The aim of this study was to assess the validity of using a single inertial sensor placed on the lower back compared to a reference system in identifying individual steps and quantifying their duration in patients with HSP while walking. The use of such a validated single-sensor approach during the in-clinic assessment of gait could augment the clinical assessment and gait monitoring of HSP patients. Excellent agreement was found between the single-sensor approach and the reference system, confirming the usability of the proposed approach in patients with HSP.

Since patients with HSP usually show a reduced walking speed due to spasticity in the lower limbs [1], weakened step-related peaks are expected in the lower-back acceleration signals [12]. Therefore, adopting an approach that uses different filters based on the peculiar feature of a given signal, while improving the signal-to-noise ratio and enhancing steps-related peaks, enabled excellent initial contact detection (mean F1 score of 0.94) in the analysed study cohort. The performances of the method presented in this study are indeed similar to those found in a previous validity study with people with HSP [30] where the stride time was instead estimated from the data measured by two IMUs placed on the feet and compared to the data from a pressure sensitive walkway while patients performed 10-metre walking tests. The performance metrics were similar to those observed in [30], with F1 score values of 0.94 ± 0.00 (current study: 0.94 [IQR: 0.86, 0.97]), where ten HSP patients (60% walking aid users) with similar age (58 ± 7 years) and SPRS scores (19.0 ± 7.5) were included. Therefore, the single-sensor approach used in the current study, which further limits patients’ burdens, has similar validity to a system consisting of two IMU sensors placed on the feet when used in an HSP cohort. Similar to [30], the highest step duration errors were observed in patients showing higher disability scores.

As expected, the performance metrics of the algorithms declined when applied to the data from patients using a walking aid, but not significantly. The lowest F1 scores (<0.7) were observed in two participants using bilateral walking aids (one used two crutches and the other used a rollator) and showing either long step time (>1 s) or high step time variability. The lower positive predicted values observed in the tests performed by patients using a walking aid indicate that more $IC_{IMU}$ were estimated than those that were actually performed. Extra $IC_{IMU}$, based on zero-crossings in the acceleration signal, could exist due to extra accelerations caused by the movement of the aid.

The detection of initial contacts is the first algorithmic step required to quantify various spatio-temporal parameters, such as cadence, step symmetry, gait variability, step time, stride time, etc., which should provide additional clinical value for assessing the disease progression in HSP, especially overcoming what could be mainly derived from subjective observational gait analysis [2] and even in-clinic timed walking tests. Overall, the proposed approach detected delayed foot-to-ground contacts (0.03 s, bias) compared to the reference events. Such delays were even more prominent when people used a walking aid (bias of 0.04 s vs. 0.02 s for the non-walking aid users). These results are expected and aligned with the findings from previous studies where a single-sensor approach was adopted in healthy participants or people with conditions affecting their mobility [31,32]. The absolute error in the IC detection was significantly higher in the tests in which the patients used a walking aid compared to the tests in which the patients did not use a walking aid, suggesting that the errors in identifying an IC with an IMU significantly increased when the patients used a walking aid. However, this difference between the groups was no longer present when the average step time over the walking tests was compared. Further caution should be used when comparing longitudinal data in which patients started adopting a walking aid as a consequence of disease progression.

While the step time can discriminate between patient groups and exhibits a consistent relationship with the measures of severity and lower-extremity function, it is not yet clear what constitutes a clinically meaningful change [33]. Therefore, future work to establish the clinical importance of longitudinal changes in the step time should consider whether the difference is within the error boundaries of the proposed approach.

The limitations of this study included the small cohort size. The results presented here are based on 13 patients with a wide range of HSP severity. As HPS is a group of heterogeneous neurodegenerative disorders underpinned by over 80 different genetic alleles, different patterns of gait impairment can exist with a spectrum of severity and rates of progression. It was not surprising that this spectrum of the gait phenotype became apparent within our current data, with one of the participants displaying longer step times (>1.5 s) compared to the other participants (<1 s). Without a larger clinical validation, it is unclear if this participant was an outlier. Longer step times could indeed occur due to the use of walking aids since the patient is able to lean on the assistive device, which allows a longer duration of the lifting of their foot (i.e., the swing phase). Future research should look into the effect of the severity of the disease on the error but also whether there are any genotype–gait phenotype associations. Moreover, since increased trunk movements can be observed also in the early phases of the disease [34,35,36,37], the adoption of a lower-back sensor will grant the possibility to collect additional trunk information to further characterise mobility in people with HSP.

## 5. Conclusions

Based on these results, the use of the proposed methodology and a lower-back single-sensor (IMU) approach provides reliable step duration data when objectively quantifying people with HSP while timed walking tests are performed during their in-clinic examinations. No significant differences were found between the participants who did and did not use a walking aid when calculating the average step time. Nevertheless, mean differences and limits of agreement for the step time errors reported here should be considered when interpreting the findings to rule out the presence of a systematic error.

Our proposed approach has the advantage of minimizing both the patients’ and assessors’ burdens whilst enabling future studies of in-clinic longitudinal gait assessments to characterise gait modifications associated with HSP. Moreover, this approach will help the development of predictive models of ambulatory natural history, support clinical trial design, and provide outcome measures to evaluate the efficacy of novel treatments in HSP.

## Figures and Tables

**Table 1:** Anthropometric patients’ characteristics and their clinical characteristics.

Parameter	Mean ± SD/Median (Quartile 1, Quartile 3)
Age (years)	54 ± 15
Height (cm)	177 ± 7
Body mass (kg)	83 ± 17
Spastic Paraplegia Rating Scale (SPRS)	19.5 (14.8, 22.0) *
Modified Ashworth Scale (MAS)	8.0 (6.0, 12.0)
Scale for the Assessment and Rating of Ataxia (SARA)	10.0 (2.0, 11.8)
Gender	3 females, 10 males
SPG mutation	SPG4: 3 patients SPG7: 9 patients Other: 1 patient

* n = 12 as a score not available for one patient.

## Data Availability

The data presented in this study are available upon reasonable request from the corresponding author. The data are not publicly available due to other ongoing analyses and publications.
